# Supplementary material for: Clinical Utility of Liquid Biopsy to Detect BRAF and NRAS Mutations in Stage III/IV Melanoma Patients by Using Real-Time PCR
Source: Cancers (Basel). 2022 Jun 21;14(13):3053. doi: 10.3390/cancers14133053 (PMC9265107; doi:10.3390/cancers14133053)
Supplement: Supplementary file 1 [file cancers-14-03053-s001.zip › cancers-1718720-supplementary.pdf]

cancers

MDPI

Article

Clinical Utility of Liquid Biopsy to Detect BRAF and NRAS Mutations in Stage III/IV Melanoma Patients by Using Real-Time PCR

S1. Supplementary Method

S1.1. Tissue Analysis Using NGS

Analyses of formalin-fixed paraffin-embedded (FFPE) tissue specimens were all performed in the Pathology Service of University of Campania "Luigi Vanvitelli" using Next Generation Sequencing (NGS). An appropriate formalin-fixed paraffin-embedded (FFPE) tissue block was selected for each case. Four unstained FFPE tissue sections were cut at 10 µm each for DNA extraction. DNA was obtained using the QIAamp®DNA FFPE kit Tissue (Qiagen, Duesseldorf, Germany), according to the manufacturer's instructions. Extracted DNA was eluted in 20 or 30 µL of elution buffer and then DNA was quantified by a Qubit® 2.0 Fluorometer (Life Technologies, Singapore) using the Qubit® dsDNA HS Assay kit, according to the manufacturer's recommendations. The extracted DNA was stored at -20°C. The Ion Torrent Personal Genome Machine (PGM) technology allows the massive parallel sequencing of DNA libraries, of several different samples, using an approach based on the PH variations that occur at the moment of incorporation of the single deoxyribonucleotide into the reaction catalyzed by the DNA polymerase. Around 10 ng of DNA was used to prepare the sequencing libraries. The libraries were prepared with the IonAmpliSeq™ Library kit 2.0 (Thermo Fisher Scientific, Carlsbad, CA, USA) and with primer pool: IonAmpliSeq Colon and Lung Cancer Research Panel v2, which analyzes 504 mutational hotspots and targeted regions in 22 genes, including BRAF and NRAS. Amplified products were purified with Agencourt AMPure XP beads (Beckman Coulter Genomics, HighWycombe, UK). Concentrations of amplified and bar-coded libraries were measured using the Qubit ® 2.0 Fluorometer (Life Technologies, Singapore) and the Qubit® dsDNA HS Assay kit (Life Technologies, Singapore). DNA libraries were stored at -20°C. The libraries were clonally amplified on Ion Sphere™ particles after dilution of the libraries to 100 pM. Template preparation was performed with the IonOneTouch™2 System (Thermo Fisher Scientific, Carlsbad, CA, USA), an automated system for emulsion PCR, recovery of Ion Sphere™ particles and enrichment of template-positive particles. The Ion Sphere™ particles coated with template were applied to the semiconductor chip. A short centrifugation step was conducted to allow the spherical particles to be deposited into the chip wells. Finally, sequencing was carried out using Ion 316™chips on the Ion Personal Genome Machine System (PGM™, Thermo Fisher Scientific) using the Ion PGM™ Hi-Q view Sequencing kit v2 (Thermo Fisher Scientific, Carlsbad, CA, USA). The Torrent Suite Software v.4.0.2 (Life Technologies) was used to assess run performance and data analysis. Integrative Genomics Viewer (IGV v 2.2, Broad Institute) was used for visual inspection of the aligned reads. Sequencing data were analyzed using Ion Reporter software (https://ionreporter.lifetechnologies.com/) and further filtered through quality checking. We selected all single nucleotide variants (SNVs) in the studied genes resulting in a non-synonymous amino acid change, or a premature stop codon, and all short indels resulting in either a frameshift or insertion/ deletion of amino acids. All SNVs were analyzed for previously reported hotspot mutations (somatic mutations reported in COSMIC database (cancer.sanger.ac.uk) and novel variations, i.e., new mutations detected by NGS but not reported in either COSMIC or db SNP databases.

S1.2. Plasma Collection

At least 6 mL of whole blood was collected by standard procedure peripheral vein blood draw, using Vacutainer® with EDTA as anticoagulant (K2EDTA, purple cap, catalog #367863, Becton Dickinson). Plasma was separated through two different centrifugation steps (the first at room temperature for 10 min at 1500x g and the second at 2000x g for the same time and temperature).

S1.3. Idylla™ Analysis

1 mL of fresh plasma was added to the specific cartridge for automated analysis. ct NRAS/BRAF cartridges were used. The mutations detected by the cartridges are: NRAS exon 2 (G12 > C/S/A/D/V, G13 > D/V/R), exon 3 (A59 > T, Q61 > K/L/R/H), exon 4 (K117 > N, A146 > T/V), BRAF V600 > E/D/K/R. The limit of detection of the Idylla™ platform for cfDNA is set at about 10,000 copies of WT DNA (about 30 ng) per milliliter. At the end of the run the platform displays an automated report that includes the followings: validity of the run (in case no DNA is detected, the test is considered invalid), presence or absence of mutation(s), type of mutation(s) detected, quantitation cycle (Cq) of the total BRAF/NRAS, Cq of the specific mutation(s) detected. Concerning BRAF mutational status, results are provided as V600E/E2/D or V600K/R/M, without discriminating which specific mutation is detected in patient's plasma sample; the same occur for NRAS Q61R/K mutations. No specific training is required in order to interpret the test result.

Cq is the cycle number at which the amplified product of PCR - specifically, BRAF-V600/NRAS mutant copies - accumulates to yield a detectable fluorescence signal by the instrument. Circulating mutational fraction (CMF) represents the percentage of mutated DNA over the internal control, calculated as follows: [2^(-ΔCq)] X100, where ΔCq is the difference between the Cq of the mutation and the mean Cq of the internal control, obtained for each positive test.

S1.4. Inclusion and Exclusion Criteria

1.4.1. Baseline Cohort

Inclusion criteria:

1) Male or female aged ≥ 18 years of age.

2) Patients with a diagnosis of locally advanced or metastatic melanoma with known BRAF-V600 (V600E/K/D/M/R) or NRAS exon 2 (G12C/S/A/D/V, G13D/V/R), exon 3 (A59 T, Q61K/L/R/H), exon 4 (K117N, A146T/V) mutation.

3) Measurable disease at the time of plasma collection (according to RECIST 1.1 criteria).

4) Candidate to receive immunotherapy (anti-PD-1) or targeted therapy (BRAFi+MEKi) for locally advanced or metastatic disease.

5) Available tumor tissue sample(s) for immunohistochemical and molecular analysis.

6) Signed informed consent form.

Exclusion criteria:

1) Patients younger than 18 years old.

2) Previous treatment for locally advanced or metastatic melanoma

3) History of another malignancy within 5 years or current 2nd primary malignancy.

4) ECOG PS ≥ 2.

5) Patients that have not signed informed consent.

1.4.2 Non-Baseline Cohort

Inclusion criteria:

1) Male or female aged ≥ 18 years of age.

2) Patients with a diagnosis of locally advanced or metastatic melanoma with known BRAF-V600 (V600E/K/D/M/R) or NRAS exon 2 (G12C/S/A/D/V, G13D/V/R), exon 3 (A59 T, Q61K/L/R/H), exon 4 (K117N, A146T/V) mutation.

3) Measurable disease at the time of plasma collection (according to RECIST 1.1 criteria).

4) Patients already receiving immunotherapy (anti-PD-1) or targeted therapy (BRAFi+MEKi) as first line for locally advanced or metastatic disease.

5) Available tumor tissue sample(s) for immunohistochemical and molecular analysis.

6) Signed informed consent form.

Exclusion criteria:

1) Patients younger than 18 years old.

2) ≥ 1 previous treatment for locally advanced or metastatic melanoma

3) History of another malignancy within 5 years or current 2nd primary malignancy.

4) ECOG PS ≥ 2.

5) Patients that have not signed informed consent.

1.4.3 Resected Cohort

Inclusion criteria:

1) Male or female aged ≥ 18 years of age.

2) Patients with a diagnosis of resected melanoma with known BRAF-V600 (V600E/K/D/M/R) or NRAS exon 2 (G12C/S/A/D/V, G13D/V/R), exon 3 (A59 T, Q61K/L/R/H), exon 4 (K117N, A146T/V) mutation.

3) Stage III at diagnosis

4) Candidate to receive adjuvant immunotherapy (anti-PD-1) or adjuvant targeted therapy (BRAFi+MEKi)

5) Available tumor tissue sample(s) for immunohistochemical and molecular analysis.

6) Signed informed consent form.

Exclusion criteria:

1) Patients younger than 18 years old.

2) Previous treatment for resected melanoma

3) History of another malignancy within 5 years or current 2nd primary malignancy.

4) ECOG PS ≥ 2.

5) Patients that have not signed informed consent.

Table S1. All positive qPCR results, in relation to time of plasma collection.

Pt n°	Time of plasma collection	Previous therapy	qPCR result	Cq	CMF (%)	LDH (ratio)	SoD (mm)
A1	Baseline	No	BRAF V600E/D	44.73	0.237	2.97*	60
A2	Baseline	No	BRAF V600K/R	52.96	0.004	0.65	50
A3	Baseline	No	BRAF V600K/R	45.16	0.111	1.02	70
A4	Baseline	No	BRAF V600E/D	44.98	0.234	0.68	46
A5	Baseline	No	BRAF V600E/D	52.65	0.008	2.03*	85
A6	Baseline	No	NRAS Q61R/K	53.13	0.0004	0.65	97
A11	Baseline	No	BRAF V600K/R	47.2	0.249	1.23	45
A13	Baseline	No	BRAF V600E/D	41.29	5.441*	0.51	65
A15	Baseline	No	NRAS Q61R/K	44.95	0.051	0.68	30
A17	Baseline	No	BRAF V600K/R	50.08	0.034	0.76	125
A18	Baseline	No	BRAF V600E/D	53.4	0.002	0.79	42
A21	Baseline	No	BRAF V600E/D	36.94	0.639*	6.46*	75
A22	Baseline	No	BRAF V600E/D	51.17	0.011	0.5	40
A23	Baseline	No	BRAF V600E/D	49.46	0.0003	0.66	38
A24	Baseline	No	BRAF V600E/D	51.05	0.001	0.77	10
A29	Non-baseline	Anti-PD-1	BRAF V600E/D	52.54	0.0003	0.73	45
A30	Non-baseline	Adjuvant anti-PD-1	BRAF V600E/D	41.47	0.572*	3.24*	70
A7	At PD	TT	BRAF V600R/K	46.88	0.018	0.88	15
A11	6-months	TT	BRAF V600R/K	55.28	0.0008	0.72	90

CMF, circulating mutational fraction; Cq, quantitation cycle; LDH, lactate dehydrogenase (expressed as LDH value / upper limit of normal LDH); qPCR, quantitative PCR; SoD, sum of lesion diameters; TT, targeted therapy. * outlier values.

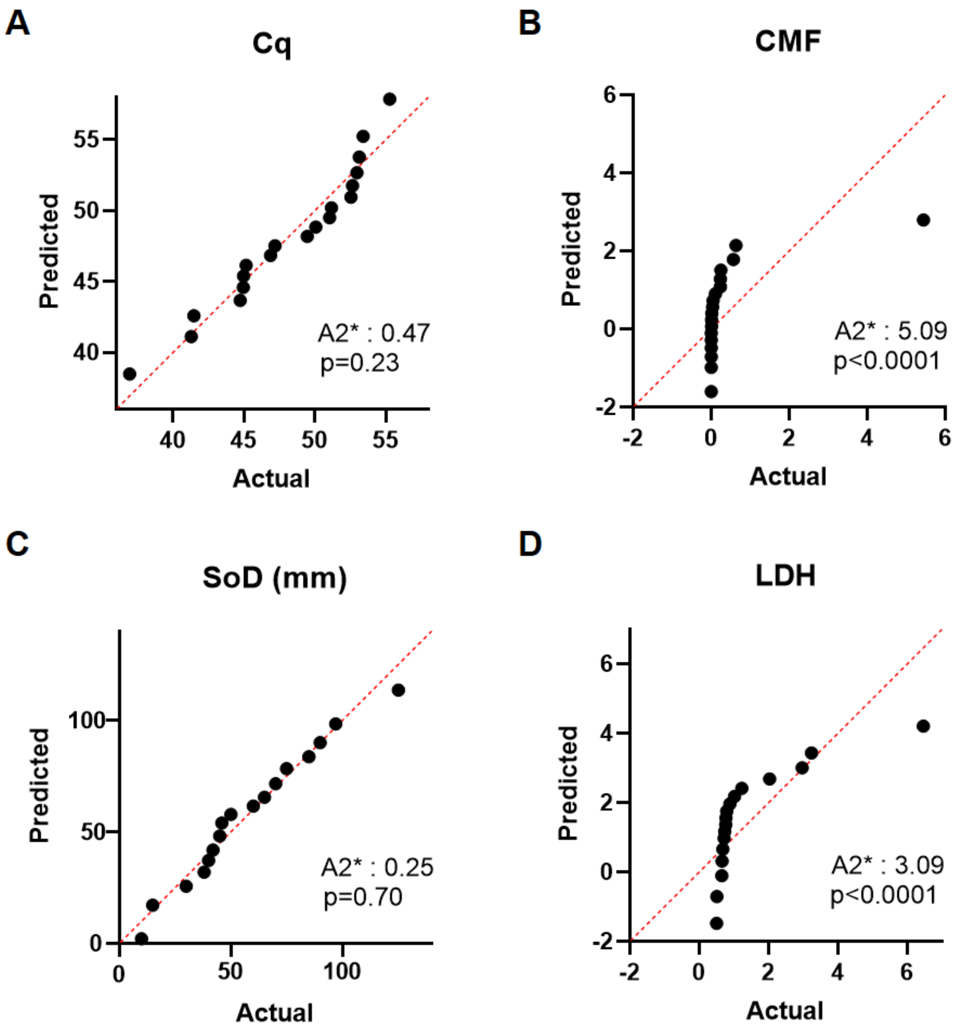

Figure S1. Normality distribution (value in Table 1): normal QQ plot for Cq (A), CMF (circulating mutational fraction) (B), SoD (sum of lesion diameters) (C), and LDH (lactate dehydrogenase, expressed as LDH value / upper limit of normal LDH) (D) with their respective Anderson-Darling test results (A2*) and p values.

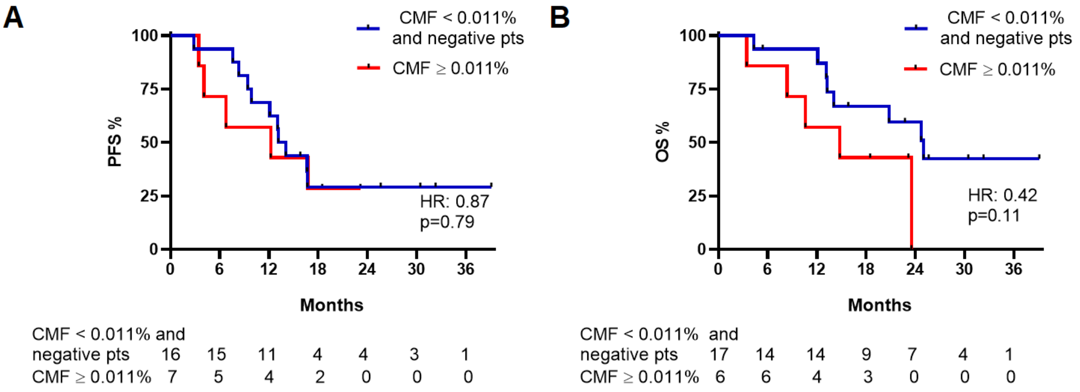

Figure S2. Progression free survival (PFS) and overall survival (OS) according to CMF values. PFS (A) and OS (B) in baseline patients with CMF lower than 0.011% (including in this group patients with no mutation detected by the instrument, see text for details) and patients with CMF value equal to or higher than 0.011%.

N, patients with no detectable mutation at baseline. CMF, circulating mutational fraction; HR, hazard ratio; PFS, progression free survival.

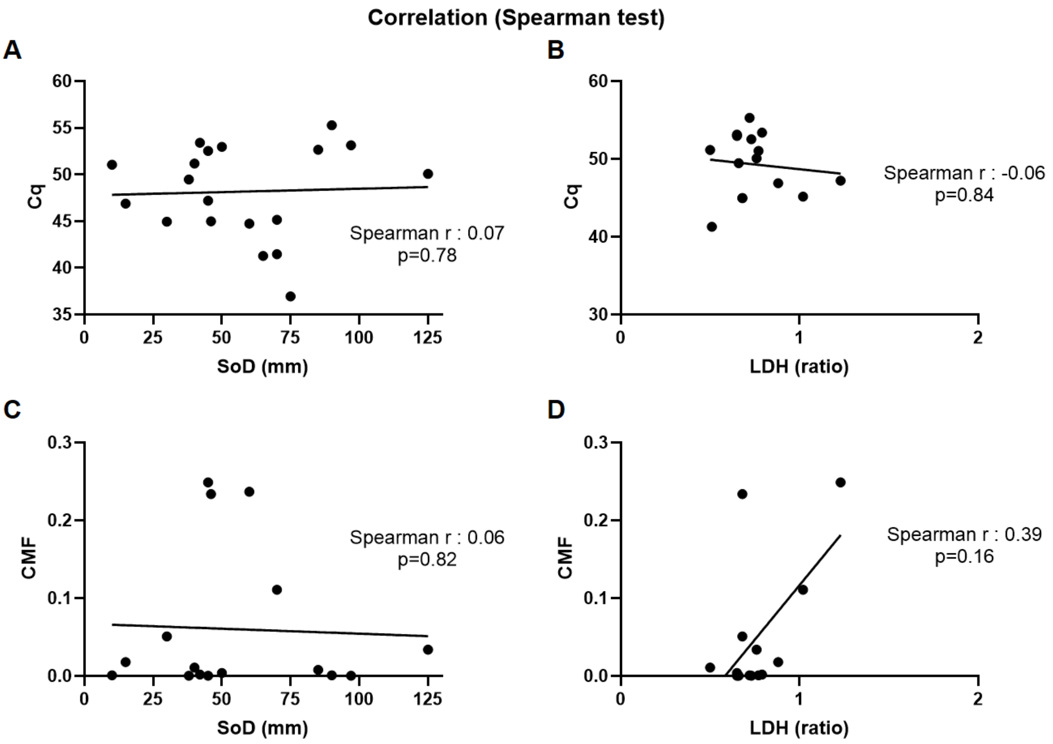

Figure S3. Spearman correlation test between Cq and SoD (A), Cq and LDH ratios (B), CMF and SoD (C), CMF and LDH ratios (D).

CMF, circulating mutational fraction; SoD, sum of lesion diameters.
